# ANTI-INFLAMMATORY AND METABOLIC EFFECTS OF *COFFEA CANEPHORA* EXTRACT IN DIABETIC RATS: INSIGHTS FROM TNF-Α REDUCTION AND INSULIN SENSITIVITY INDICES

**DOI:** 10.21010/Ajidv19i2S.5

**Published:** 2025-10-17

**Authors:** Jekson Martiar Siahaan, Endy Juli Anto, Tengku Muhammad Fauzi, Sumihar MR Pasaribu, Hana Isal Salina Ginting, Friska Ernita Sitorus, Hariati Hariati, Firdaus Fahdi, Peny Ariani, Untung Sujianto, Rostime Hermayerni Simanullang, Suryati Sinurat, Jadeny Sinatra, Hadyanto Lim

**Affiliations:** 1 Department of Physiology, Faculty of Medicine, Institut Kesehatan Deli Husada Deli Tua, Indonesia; 2 Department of Parasitology, Faculty of Medicine, Universitas Methodist Indonesia, Medan, Indonesia; 3 Department of Biochemistry, Faculty of Medicine, Universitas Methodist Indonesia, Medan, Indonesia; 4 Department of Biochemistry, Faculty of Medicine, Institut Kesehatan Deli Husada Deli Tua, Indonesia; 5 Department of Parasitology, Faculty of Medicine, Institut Kesehatan Deli Husada Deli Tua, Indonesia; 6 Department of Medical-Surgical Nursing, Faculty of Nursing, Institut Kesehatan Deli Husada Deli Tua, Indonesia; 7 Department of Pharmacy, Faculty of Pharmacy, Institut Kesehatan Deli Husada Deli Tua, Indonesia; 8 Faculty of midwifery, Institut Kesehatan Deli Husada Deli Tua, Indonesia; 9 Department of Nursing, Faculty of of Medicine, Universitas Diponegoro, Semarang, Indonesia; 10 Department of Nursing, Faculty of Nursing, Universitas Murni Teguh, Medan, Indonesia; 11 Department of Public Health, Faculty of Medicine, Universitas Methodist Indonesia, Medan, Indonesia; 12 Department of Anesthesiology, Faculty of Medicine, Universitas Methodist Indonesia, Medan, Indonesia; 13 Department of Pharmacology, Faculty of Medicine, Universitas Methodist Indonesia, Medan, Sumatra Utara, Indonesia.

**Keywords:** Coffea canephora var. robusta, Glucose Homeostasis, Antiinflammatory

## Abstract

**Background:**

Type 2 diabetes mellitus (T2DM) is a chronic metabolic disorder characterized by Insulin resistance, hyperglycemia, and systemic inflammation. *Coffea canephora* var. *robusta* contains bioactive compounds with potential antihyperglycemic and anti-inflammatory effects. This study aimed to evaluate the metabolic and inflammatory effects of its ethanolic extract in a rat model of T2DM.

**Materials and Methods:**

Forty-four male Wistar rats were randomized into 11 groups, including normal, diabetic controls, and treatment groups receiving ethanolic or ethyl acetate extracts (100–400 mg/kgBW). T2DM was induced using a high-fat diet and streptozotocin ± nicotinamide. Outcomes measured were fasting blood glucose (FBG), glucose-lowering percentage (%GL), fasting insulin resistance index (FIRI), quantitative insulin sensitivity check index (QUICKI), and TNF-α levels.

**Results:**

The 400 mg/kgBW ethanolic extract group showed a significant reduction in FBG (*p* = 0.017), percentage of glucose lowering (*p* = 0.000), TNF-α levels (*p* = 0.000), and body weight (*p* = 0.000) compared to diabetic controls, indicating improved metabolic regulation.

**Conclusion:**

The ethanolic extract of *C. canephora* improves glycemic control, enhances insulin sensitivity, and reduces inflammation in T2DM rats, supporting its potential for future clinical validation.

## Introduction

In 2024, the global number of individuals living with diabetes reached approximately 588.7 million. This figure is projected to increase by 45% by the year 2050, with an estimated total of over 850 million people affected worldwide. The global health expenditure associated with diabetes has also escalated significantly, reaching USD 1 trillion—a 338% increase over the past 17 years (International Diabetes Federation, 2025). This metabolic disorder is characterized by chronic hyperglycemia due to insulin resistance and progressive β-cell dysfunction (Siahaan, *et al.*, 2019; Siahaan *et al.*, 2020). Despite current therapeutic regimens, including insulin sensitizers and secretagogues, long-term glycemic control remains suboptimal in many patients, often necessitating the exploration of alternative and complementary interventions (Siahaan, *et al.*, 2019). Recent attention has been directed toward the role of natural products in restoring glucose homeostasis and preserving pancreatic β-cell function. Among various candidates, *Coffea canephora* var. *robusta* (Robusta coffee) has attracted scientific interest due to its rich phytochemical profile, including chlorogenic acids, caffeine, and diterpenes, which have been linked to improved insulin sensitivity and antioxidant properties (Ihara, *et al.*, 2023; Nguyen, *et al.*, 2024). Evidence suggests that chronic consumption of coffee may reduce the risk of T2DM, though the underlying mechanisms, particularly at the cellular and molecular levels, remain incompletely understood (Reis, *et al.*, 2019; Kolb, *et al.*, 2021; Mohamed, *et al.*, 2024).

Experimental models mimicking human T2DM, such as high-fat diet (HFD) combined with streptozotocin-nicotinamide (STZ-NA) administration, are widely used to study β-cell dysfunction in a physiologically relevant context (Siahaan, *et al.*, 2019; Siahaan *et al.*, 2020). This model induces insulin resistance followed by partial β-cell damage, enabling the assessment of interventions targeting both insulin action and β-cell regeneration. In this context, evaluating biomarkers related to glucose homeostasis—such as fasting blood glucose, insulin levels, and insulin resistance index (e.g., HOMA-IR, FIRI, QUICKI)—is critical for determining metabolic improvements (Kolb, *et al.*, 2021; Mohamed, *et al.*, 2024). In addition, the assessment of pancreatic β-cell proliferation is essential to elucidate whether a compound exerts regenerative effects. Several recent studies have explored molecular pathways involved in β-cell proliferation, including cell cycle regulators such as Cyclin D1, Cyclin D2, and CDK4/6 via pathways like Akt–mTOR, GSK-3β–β-catenin, and STAT3/5–ICA512 [10]. While most prior research assessed expression levels using advanced molecular techniques, an emerging alternative includes quantifying Cyclin D1 activity using enzyme-linked immunosorbent assay (ELISA), offering a practical and reliable method for biomarker evaluation in preclinical studies (Luo, *et al.*, 2020).

Despite increasing evidence on the metabolic and anti-inflammatory potential of *Coffea canephora* (Robusta coffee), few studies have comprehensively evaluated its effects on glycemic control and systemic inflammation in type 2 diabetes mellitus (T2DM). This study aims to assess the anti-inflammatory and metabolic benefits of *Coffea canephora* extract in HFD–STZ–NA-induced diabetic rats by evaluating changes in body weight, fasting blood glucose, serum insulin levels, TNF-α concentrations, and insulin sensitivity indices (FIRI and QUICKI). The findings are expected to offer new insights into the dual role of Robusta coffee in modulating inflammatory responses and improving insulin sensitivity in T2DM management.

## Materials and Methods

### Extraction of Coffea canephora var. robusta

The extraction process of *Coffea canephora* var. *robusta* beans followed a cold maceration protocol using 95% ethanol as the solvent. Initially, mature beans were thoroughly washed with distilled water to remove dirt and surface contaminants, followed by air drying under shade conditions to prevent photodegradation of phytochemicals. Once dried, the beans were finely ground using a mortar and pestle to produce a uniform powder.

A total of 50 grams of the powdered beans was accurately weighed and transferred into a 250 mL Erlenmeyer flask. Then, 200 mL of 95% ethanol was added to the flask. The mixture was mixed and left to macerate and allowed to macerate at ambient room temperature (±25°C) for 24 hours to ensure optimal extraction of bioactive compounds. After maceration, the solution was filtered using Whatman No. 1 filter paper and a glass funnel to remove plant debris.

The resulting filtrate, which contained the crude ethanol extract, was concentrated using a rotary evaporator under reduced pressure at a controlled temperature of 40°C to avoid degradation of heat-sensitive constituents. The semi-solid residue obtained was then redissolved in distilled water to prepare the final working extract. The extraction yield (%) was determined by dividing the dry weight of the extract obtained by the initial weight of the powdered beans and multiplying by 100. The prepared extract was stored in airtight amber containers under cool, dry conditions until further use in animal experiments.

### Animal Model and Experimental Design

Male albino rats (*Rattus norvegicus*), aged 8–10 weeks and weighing approximately 180–200 g, were used in this study. All animals were housed under controlled environmental conditions with a 12-hour light/dark cycle, standard pellet diet, and water *ad libitum*. The study protocol received ethical approval and complied with institutional animal care guidelines. The rats were randomly assigned into eleven groups (n = 4 per group), based on Federer’s formula for experimental design. The grouping was as follows (Sinaga, *et al.*, 2025):

- **Group A (Normal Control):** Healthy rats with no treatment, receiving a standard diet and water *ad libitum*
- **Group B (Positive Control):** Induced with a high-fat diet (HFD), nicotinamide (110 mg/kg body weight, i.p.), and streptozotocin (STZ, 45 mg/kg body weight, i.p.)
- **Group C:** Induced with HFD and STZ (without nicotinamide).
- **Group D:** Induced with HFD and nicotinamide (without STZ).
- **Group E:** Treated with ethanolic extract of *Coffea canephora* var. *robusta* at 100 mg/kg body weight, administered orally (p.o.)
- **Group F:** Treated with ethanolic extract of *Coffea canephora* var. *robusta* at 200 mg/kg body weight, p.o.
- **Group G:** Treated with ethanolic extract of *Coffea canephora* var. *robusta* at 400 mg/kg body weight, p.o.
- **Group H:** Treated with ethyl acetate fraction of *Coffea canephora* var. *robusta* at 100 mg/kg body weight, p.o.
- **Group I:** Treated with ethyl acetate fraction of *Coffea canephora* var. *robusta* at 200 mg/kg body weight, administered orally (p.o.)
- **Group J:** Treated with ethyl acetate fraction of *Coffea canephora* var. *robusta* at 400 mg/kg body weight, p.o.
- **Group K:** Treated with metformin at 40.5 mg/kg body weight, p.o.

Induction of Type 2 Diabetes Mellitus

To establish an insulin-resistant and hyperglycemic state resembling Type 2 Diabetes Mellitus (T2DM), a two-step induction protocol was employed. First, insulin resistance was induced by daily oral administration of a high-fat diet (HFD) mixture containing quail egg yolk, coconut oil, and butter in a 1:6:3 ratio. Each rat received 4 mL of this mixture per day for a designated pre-induction period.

In the second phase, T2DM was induced by intraperitoneal injections of nicotinamide (110 mg/kg bw) followed by streptozotocin (45 mg/kg bw), once daily for 30 consecutive days. After 72 hours from the last injection, blood glucose levels were measured using a glucometer. Rats exhibiting fasting blood glucose levels exceeding 250 mg/dL were considered diabetic and included in the treatment phase.

### Treatment and Biochemical Assessment

The administration of treatments, including extracts, fractions, and metformin, was carried out once daily via oral gavage for 28 consecutive days. To ensure consistency in metabolic timing, all administrations were performed at the same time each day.

Blood samples were collected at three key time points:

- **Day 0 (H0)**: Baseline glucose levels before induction.
- **Day 1 (H1)**: Post-induction blood glucose levels to confirm diabetic status.
- **Day 28 (H28)**: Final measurements following 28 days of treatment.

The concentration of plasma insulin was measured using a commercially available ELISA kit, following the manufacturer’s protocol. This assessment was conducted to determine the functional status of pancreatic β-cells and their capacity to secrete insulin. The study was approved by the Health Research Ethics Commission, Faculty of Medicine, Universitas Methodist Indonesia, under approval number 08/KEPK-FKUMI/EC/2024.

## Results and Discussion

As shown in Table 1, the **baseline fasting blood glucose** ranged from **93.3 ± 27.9 mg/dL** (Group I) to **132 ± 29.3 mg/dL**(Group K), with no statistical difference among groups (p > 0.05). Similarly, **baseline body weight** did not differ significantly (p > 0.05), with Group F having the highest weight (230.75 ± 7.68 g) and Group C the lowest. This initial consistency confirms the appropriateness of the randomization procedure and ensures that all subjects started under comparable metabolic conditions. Baseline equivalence in both glucose and body weight is crucial in preclinical diabetes research, as it minimizes confounding variables and strengthens internal validity. When subjects begin at different metabolic baselines especially within small groups later treatment effects may be misattributed to preexisting disparities.

**Table 1:** Examination of Blood Glucose, and Body Weight

Group	Examination					
	Baseline Blood Glucose (mean±SD)	Post Induction Blood Glucose (mean±SD)	Final Fasting Blood Glucose (mean±SD)	Baseline Body Weight (mean±SD)	Post Induction Body Weight (mean±SD)	Final Body Weight (mean±SD)
A	113.5±16.29	114.75±11.84	114.75±12.6	222.25±24.636	205.25±24.432	233.00±22.076
B	107.25±18.94	262.25±9.81	294.25±103.13	212.25±26.887	238.25±37.704	318.25±69.945
C	112.75±2.50	255.75±4.34	295.5±65.32	197.75±9.069	195.75±8.884	252.25±39.255
D	109.75±2.62	254.75±6.29	210.75±76.7	218.00±19.613	244.00±22.181	227.50±22.664
E	108.75±8.61	411.5±142.82	150.75±115.24	227.50±22.008	230.00±22.390	209.50±14.663
F	117±15.34	401±10937	253.25±148.36	230.75±7.676	237.00±3.830	205.50±8.103
G	123.25±29.89	404±102.53	80.5±17.59	212.50±23.502	271.25±17.056	165.25±23.042
H	117.25±12.148	383.75±38.70	283.75±210.17	223.75±19.923	273.00±23.875	259.25±28.826
I	93.25±27.93	459±123.12	101.25±9.53	209.25±22.381	280.50±11.790	271.75±11.587
J	105.25±18.39	335±109.19	163.25±94.91	225±21.024	273.00±22.906	255.50±36.208
K	132±29.31	379.75±84.46	130.25±22.02	201.25±6.397	284.00±6.481	276.00±6.481
P- value	0.376	0.000*	0.017*	0.333	0.000*	0.000*

Ensuring homogeneity at baseline allows for more definitive attribution of observed effects to the interventions rather than to natural variability. In recent protocols for rodent metabolic studies, strict control of baseline characteristics such as fasting glucose and body mass is emphasized to reduce variability and enhance reproducibility (Benedé-Ubieto, *et al.*, 2020; Hahn & Pereira, 2024). Such standardization allows researchers to more accurately detect treatment-related changes in follow-up measures like glucose tolerance, insulin resistance indices, and inflammatory markers. Importantly, because all groups were equivalent at baseline, we can interpret the subsequent improvements observed in **Group G** (400 mg/kgBW *C. canephora* extract) as genuine treatment effects. The significant improvements in glycemic parameters (fasting glucose, %Gl), insulin sensitivity (QUICKI), insulin resistance (FIRI), and inflammation (TNF-α) are therefore likely attributable to the biological activity of **chlorogenic acids and diterpenes** present in the extract, rather than to differences in baseline status (Tamimi, *et al.*, 2024;). It is also worth noting that baseline body weight is a critical determinant of metabolic outcomes: variations in adiposity can influence insulin signaling, systemic inflammation, and energy homeostasis (An, *et al.*, 2023;). By confirming that all groups began with similar body weights, we strengthen the validity of comparisons made post-treatment. Following the induction phase using a combination of high-fat diet (HFD), streptozotocin (STZ), and nicotinamide (NA), a clear elevation in fasting blood glucose (FBG) levels was observed across all induced groups. The **highest mean FBG**was detected in **Group I (459 ± 123.12 mg/dL)**, whereas the **lowest level** was found in **Group A (114.75 ± 11.84 mg/dL)**, which served as the non-diabetic control and did not receive any induction. **Statistical analysis revealed a significant difference between groups (p < 0.005)**, indicating that the induction protocol effectively established a type 2 diabetes mellitus (T2DM) model. The hyperglycemic state seen in the induced groups aligns with the pathological features of T2DM, where insulin resistance is compounded by partial pancreatic β-cell dysfunction—a condition commonly reproduced in rodents using STZ-HFD protocols. Notably, the inclusion of nicotinamide serves to attenuate STZ-induced β-cell cytotoxicity, resulting in a partial insulin-deficient state that more closely mirrors the progressive nature of T2DM in humans (Siahaan, *et al.*, 2019; Siahaan *et al.*, 2020).

This consistent rise in post-induction glycemia, especially among the diabetic groups, serves not only as evidence of successful model development but also provides a strong foundation for assessing the therapeutic efficacy of the interventions, particularly the *Coffea canephora* extract. The significant intergroup variability observed at this stage also allows for the stratification of responses in subsequent treatment evaluations. Elevated glucose levels in diabetic groups, relative to controls, confirm pathological induction, while differences among those groups may indicate different levels of metabolic damage or an early response to treatment. At the end of the 28-day intervention, **Group B exhibited the highest mean fasting blood glucose level (294.25 ± 103.13 mg/dL)**, whereas the **lowest value was recorded in Group G (80.5 ± 17.59 mg/dL)**. The intergroup comparison revealed a **statistically significant difference (p < 0.05)**, confirming that the **oral administration of** *Coffea canephora* **var.** *robusta* **extract at 400 mg/kgBW** produced the most pronounced glycemic-lowering effect. This outcome underscores a **dose-dependent antihyperglycemic response**, with the highest tested dose yielding the greatest benefit. Such findings are in agreement with recent evidence highlighting the therapeutic role of polyphenolic compounds—particularly **chlorogenic acids**—in regulating glucose metabolism. These bioactive molecules are reported to inhibit hepatic glucose-6-phosphatase activity, attenuate intestinal glucose uptake, and enhance insulin sensitivity through modulation of insulin receptor signaling pathways (Barik, *et al.*, 2024). In addition to chlorogenic acid, *Coffea canephora* is rich in **diterpenes** such as **cafestol** and **kahweol**, which have demonstrated insulinotropic effects and protective actions on pancreatic β-cells under oxidative stress conditions. These compounds collectively contribute to a more efficient glycemic profile, especially in insulin-resistant states (Ren, *et al.*, 2024).

The marked decline in glucose levels in Group G not only supports the direct antihyperglycemic potential of the extract but also suggests possible modulation of key metabolic pathways involved in insulin sensitivity and hepatic gluconeogenesis. This interpretation is further validated by favorable changes observed in complementary indicators such as QUICKI and FIRI, both of which reflect improved insulin action (Hamed, *et al.*, 2025). Overall, these findings present compelling evidence that ***Coffea canephora* extract**, particularly at the 400 mg/kgBW dosage, holds promise as a **natural adjunct therapy in type 2 diabetes management**, offering benefits that extend beyond glycemic control to include insulin regulation and metabolic stabilization. Following the induction protocol, a **significant divergence in body weight** was observed across experimental groups (**p < 0.05**), despite initial equivalence at baseline. **Group K displayed the highest post-induction mean body weight (284.00 ± 6.48 g)**, whereas **Group C showed the lowest (195.75 ± 8.88 g)**. This variation reflects the **differential metabolic consequences** of the combined exposure to a high-fat diet (HFD), streptozotocin (STZ), and nicotinamide (NA), which are widely used to induce type 2 diabetes mellitus (T2DM) in rodent models. The reduction in body mass, particularly evident in certain diabetic groups, is consistent with the **catabolic profile** associated with STZ-induced pancreatic β-cell impairment. When insulin production is compromised, anabolic processes such as glycogen synthesis and protein accretion decline, leading to muscle wasting and weight loss. In contrast, animals that experience partial β-cell protection or insulin resistance without complete loss of insulin signaling—often due to dietary fat overload—may retain or even accumulate adipose tissue, contributing to higher post-induction weight (Guerra-Ávila, *et al.*, 2024; Brito, *et al.*, 2025). Group G showed a moderate final body weight, suggesting the extract may help prevent severe metabolic imbalance. This modulation aligns with enhanced insulin sensitivity, reduced inflammatory signaling, and improved glycemic control as evidenced by better FIRI, QUICKI, and fasting glucose—effects consistent with findings that green coffee extract upregulates adiponectin and GLUT4 while lowering HOMA-IR in obese rodent models. Moreover, varietal differences in coffee (Arabica vs Robusta) have been shown to differentially impact weight regulation and metabolic outcomes in diabetic rats, further supporting the therapeutic potential of such extracts in moderating post-induction body weight changes and enhancing metabolic resilience (Khedr, *et al.*, 2024). By the 28th day of the experimental protocol, the lowest final body weight was recorded in **Group G (165.25 ± 23.04 g)**, which received the highest dose (400 mg/kgBW) of the **ethanolic extract of** *Coffea canephora* **var.** *robusta*. This outcome was **statistically significant (p < 0.005)**, suggesting a meaningful physiological response that may extend beyond glycemic regulation. In rodent models of type 2 diabetes mellitus (T2DM), significant reductions in body weight are typically associated with insulin insufficiency, elevated catabolic activity, and skeletal muscle breakdown due to chronic hyperglycemia (Gaur, *et al.*, 2024). However, the **controlled decline in weight** observed in Group G—distinct from the more severe weight loss often seen in untreated diabetic animals—coincided with **improved insulin sensitivity**, as reflected by optimal QUICKI and FIRI values. This indicates that the weight reduction was likely **therapeutic** rather than pathological (Laker, *et al.*, 2025). Several bioactive compounds found in *Coffea canephora*, particularly **chlorogenic acids**, have been extensively studied for their roles in metabolic regulation. These molecules have been shown to suppress adipogenesis, enhance lipid oxidation, and activate **AMP-activated protein kinase (AMPK)**—a central regulator of cellular energy homeostasis. Activation of AMPK shifts metabolism toward increased fat utilization and improved glucose uptake, both of which support **healthy body composition and metabolic efficiency**. The fact that Group G showed both a **reduction in body mass** and **superior metabolic markers** compared to diabetic controls reinforces the idea that the extract has **dual therapeutic effects**: lowering blood glucose and **modulating body weight**. Unlike diabetic groups that either maintained or gained weight—potentially due to unresolved insulin resistance or lipid accumulation—the downward trend in Group G suggests a normalization of metabolic pathways rather than wasting (Vasileva, *et al.*, 2020).

Data presented in Table 2 show a significant variation in tumor necrosis factor-alpha (TNF-α) levels across groups (**p < 0.05**), with the **lowest concentration recorded in Group G**, which received the **highest dose (400 mg/kgBW)** of the ethanolic extract of *Coffea canephora* var. *robusta*. This outcome highlights the **potent anti-inflammatory activity** of the extract, particularly in comparison to untreated diabetic controls and other intervention groups.

**Table 2:** Examination of TNF α, INSULIN,QUICKI, FIRI, % Glocose Lowering

Group	Examination				
	TNF α (mean±SD)	INSULIN (mean±SD)	QUICKI (mean±SD)	FIRI (mean±SD)	%Glucose Lowering (mean±SD)
A	94.50±3.687	4.57±0.94	0.343000±0.0131403	33.9975±7.84491	0.0450±1.52636
B	195.75±38.888	2.51±1.87	0.451500±0.2082058	25.7025±23.20826	-11.250±36.06351
C	113.75±3.500	5.17±3.28	0.405000±0.1343304	53.3200±63.79187	-1.1875±31.48684
D	103.75±3.500	4.71±3.44	0.497250±0.1417565	21.2575±36.60088	28.1975±19.59732
E	93.75±3.500	0.44±0.23	0.401500±0.1103615	23.6875±14.47742	64.6850±16.69193
F	83.75±3.500	6.87±0.99	0.333000±0.0091287	45.8700±9.54351	39.5850±25.49144
G	64.25±3.304	6.62±0.94	0.559250±0.0410396	2.6050±1.33902	78.9750±8.22268
H	77.25±5.123	6.14±0.70	0.324750±0.0025000	55.8525±4.16613	25.7650±55.99923
I	84.00±3.651	3.77±2.41	0.329750±0.0180069	51.0575±20.92587	76.3825±7.91370
J	89.75±1.500	4.71±3.38	0.378500±0.1281080	53.7525±45.80888	52.4500±16.41380
K	95.75±3.594	4.58±3.69	0.415250±0.1341451	28.4450±24.55907	63.2525±14.82558
p-value	0.000*	0.024	0.081	0.229	0.000*

Tumor necrosis factor-alpha is a central mediator of chronic inflammation and is known to exacerbate insulin resistance, induce β-cell dysfunction, and accelerate the progression of metabolic disturbances in type 2 diabetes mellitus (T2DM). Therefore, the **significant suppression of TNF-α in Group G** suggests that the extract may modulate inflammatory signaling, potentially improving insulin responsiveness and glycemic control. This interpretation aligns with the favorable QUICKI and FIRI values observed in the same group (Amarachi, *et al.*, 2024).

The anti-inflammatory effect observed in this model likely stems from the bioactive constituents of *Coffea canephora*, particularly **chlorogenic acid**, **caffeic acid**, and **ferulic acid**. These compounds have been extensively reported to inhibit **NF-κB translocation**, downregulate cytokine synthesis, and counteract oxidative stress in both animal models and cellular systems of diabetes (Ouyang, *et al.*, 2024; Bao, *et al.*, 2025).

The ability of Group G to maintain lower TNF-α levels not only reflects reduced inflammatory burden but also implies **protective effects on endothelial function and tissue homeostasis**, which are essential for preventing microvascular and macrovascular complications commonly seen in diabetic patients. These findings provide compelling support for the **anti-inflammatory potential of** *Coffea canephora* as part of an integrative approach to T2DM therapy.

In this study, **Group G showed the highest QUICKI score** among all groups, indicating improved insulin sensitivity. However, the associated **p-value of 0.081** suggests that the difference did not meet the conventional threshold for statistical significance (typically p < 0.05). It is essential, especially in preclinical studies with relatively small sample sizes, to distinguish between statistical significance and **biological importance**. Despite the lack of statistical significance, the elevated QUICKI in Group G—alongside **notable reductions in fasting glucose and FIRI**—strongly suggests a genuine improvement in insulin responsiveness. QUICKI is a sensitive and well-established marker that inversely correlates with insulin resistance and reflects metabolic responsiveness in insulin-target tissues such as **skeletal muscle and adipose tissue** (Kim, *et al.*, 2025).

The absence of a statistically significant result may be attributed to factors such as **biological variation between animals**, **a limited number of subjects**, or **overlapping standard deviations** across groups, all of which can mask true physiological differences. In animal models, especially those with small sample sizes, **p-values approaching 0.05 (e.g., p = 0.081)** often indicate **biologically meaningful trends**, especially when supported by consistent changes in related markers—such as reduced TNF-α and improved FIRI values observed in the same group. The observed metabolic benefits in Group G are most likely mediated by the **bioactive components of Coffea canephora**, particularly **chlorogenic acid**, **caffeic acid**, and **ferulic acid**. These polyphenols are known to activate **AMP-activated protein kinase (AMPK)**, downregulate pro-inflammatory signaling via **NF-κB**, and enhance **GLUT4 translocation**, collectively contributing to improved glucose uptake and insulin signaling. Although the statistical threshold was narrowly missed, the **consistent biological pattern observed across multiple parameters** reinforces the hypothesis that Coffea canephora extract exerts **insulin-sensitizing effects**. Further research with **larger sample sizes** and **extended treatment durations** is warranted to confirm and strengthen the observed trends. Although **Group G** showed the **lowest mean value for the Fasting Insulin Resistance Index (FIRI)**, indicating a potential improvement in insulin responsiveness, the difference did not attain **statistical significance (p > 0.05)**. A comparable pattern was observed for the **QUICKI index**, which, despite being the **highest** in Group G, also failed to demonstrate a statistically significant distinction when compared to other groups. These findings highlight the need for interpreting results based not only on statistical thresholds but also on **biological consistency and relevance** (Ong, *et al.*, 2012; Khedr, *et al.*, 2024).

FIRI remains a widely accepted index for evaluating **insulin resistance under fasting conditions**, where higher values are associated with greater insulin resistance and metabolic dysfunction (Siahaan, *et al.*, 2019; Siahaan *et al.*, 2020). In the present study, **Group J exhibited the highest FIRI score**, which is in line with its elevated fasting glucose levels and poor glycemic parameters, indicating significant metabolic deterioration. Conversely, animals in **Group G**, administered 400 mg/kgBW of *Coffea canephora*extract, showed the **lowest FIRI**, suggesting that the extract may have supported improved insulin activity and glycemic balance.

The lack of statistical significance observed in both FIRI and QUICKI may stem from several plausible factors. One possibility is **biological variability** in individual response to treatment, which can reduce the ability to detect group-level differences. Additionally, the **sample size** employed in each group may not have been large enough to generate the statistical power required to reach significance, even in the presence of real biological improvements.Furthermore, it is important to recognize that both FIRI and QUICKI are **indirect measures**, computed from glucose and insulin concentrations. Even slight fluctuations in these underlying variables can lead to relatively high dispersion in calculated indices, particularly in small animal models. This statistical noise may obscure meaningful physiological differences between experimental groups. Nevertheless, from a biological perspective, the overall direction and alignment of the findings—such as the **reduction in fasting glucose and FIRI**, the **increase in QUICKI**, and **lower TNF-α concentrations** in Group G—offer compelling evidence of enhanced insulin sensitivity. These outcomes collectively strengthen the inference that the **extract improved metabolic health**, despite not all parameters reaching statistical significance. This supports the broader view in biomedical research that **biological relevance should complement statistical interpretation**, especially in exploratory or preclinical studies. The proposed mechanism behind these effects lies in the **phytochemical content of robusta coffee**, particularly **chlorogenic acid**, **cafestol**, and **kahweol**, which have demonstrated the ability to **activate AMPK**, reduce inflammatory signaling through **NF-κB inhibition**, and facilitate **insulin receptor activity** in target tissues such as liver and skeletal muscle (Gu *et al.*, 2023; Li, *et al.*, 2025). In summary, while formal significance thresholds were not achieved, the **consistent pattern of metabolic improvements observed in Group G** substantiates the therapeutic potential of *Coffea canephora* extract in modulating insulin function and managing hyperglycemia in type 2 diabetes mellitus (T2DM) models.

## Conclusion

This study demonstrated that oral administration of ethanolic extract of *Coffea canephora* var. *robusta* at 400 mg/kg body weight significantly improved glycemic control, insulin sensitivity, and inflammatory status in type 2 diabetic rats. These findings support its potential as a natural antidiabetic agent and warrant further investigation for clinical validation.

**Conflict of Interests:** We have No conflict of interest

**Author Contributions:** Equal contribution for all authors.

**List of Abbreviations:**
ELISA: Enzyme-Linked Immunosorbent Assay;
FBG: Fasting Blood Glucose;
FIRI: Fasting Insulin Resistance Index;
HFD: High-Fat Diet;
I.P. : Intraperitoneal;
P.O : Per Oral;
STZ-NA : Streptozotocin-Nicotinamide;
TNF-α : tumor necrosis factor-alpha;
T2DM : Type 2 Diabetes Mellitus;
QUICKI : Quantitative Insulin Sensitivity Check Index;
%GL : Percentage Of Glucose Lowering.
